# Preparation of Surface Dispersed WO_3_/BiVO_4_ Heterojunction Arrays and Their Photoelectrochemical Performance for Water Splitting

**DOI:** 10.3390/molecules29020372

**Published:** 2024-01-11

**Authors:** Xiaoli Fan, Qinying Chen, Fei Zhu, Tao Wang, Bin Gao, Li Song, Jianping He

**Affiliations:** 1 Jiangsu Key Laboratory of Advanced Structural Materials and Application Technology, School of Materials Science and Engineering, Nanjing Institute of Technology, Nanjing 211167, China; fanxl@njit.edu.cn (X.F.); chenqy18913422137@163.com (Q.C.); 13912487038@163.com (F.Z.); 2 College of Materials Science and Technology, Nanjing University of Aeronautics and Astronautics, Nanjing 210016, China; gaobin0601@nuaa.edu.cn; 3 School of Environmental Science and Engineering, Nanjing University of Information Science & Technology, Nanjing 210044, China; songli@nuist.edu.cn

**Keywords:** bismuth vanadate, tungsten oxide, heterojunction, surface dispersed heterojunction, photoelectrochemical water oxidation

## Abstract

In this work, a surface dispersed heterojunction of BiVO_4_-nanoparticle@WO_3_-nanoflake was successfully prepared by hydrothermal combined with solvothermal method. We optimized the morphology of the WO_3_ nanoflakes and BiVO_4_ nanoparticles by controlling the synthesis conditions to get the uniform BiVO_4_ loaded on the surface of WO_3_ arrays. The phase composition and morphology evolution with different reaction precursors were investigated in detail. When used as photoanodes, the WO_3_/BiVO_4_ composite exhibits superior activity with photocurrent at 3.53 mA cm^−2^ for photoelectrochemical (PEC) water oxidation, which is twice that of pure WO_3_ photoanode. The superior surface dispersion structure of the BiVO_4_-nanoparticle@WO_3_-nanoflake heterojunction ensures a large effective heterojunction area and relieves the interfacial hole accumulation at the same time, which contributes to the improved photocurrents together with the stability of the WO_3_/BiVO_4_ photoanodes.

## 1. Introduction

Photoelectrochemical (PEC) water splitting provides a promising strategy to relieve global energy demands and environmental problems as it can harvest solar energy and convert it into chemical energy in the form of a reaction product of hydrogen [1,2,3,4]. In a PEC system, the photoanode, where the oxygen evolution reaction (OER) takes place, suffers from sluggish kinetics and appears to be a rate-limiting step [5,6]. It is of great significance to searching for effective photoanodes.

Among the promising photoanode materials, WO_3_, which is featured with electron mobility of 10 cm^2^ V^−1^ s^−1^, electron diffusion length of ~500 nm and hole diffusion distance of ~150 nm [7,8], has significant advantages in charge carrier transport characteristics over other common semiconductors, such as TiO_2_ [9,10,11], ZnO [12,13] and α-Fe_2_O_3_ [14,15,16]. However, although WO_3_ can respond to visible light to some extent, the large band gap still leads to limited visible light absorption [17,18]. Thus, the theoretical solar to hydrogen (STH) efficiency of WO_3_ is only about 4.5% [19]. BiVO_4_ possesses a narrow band gap of ~2.4 eV, which can absorb a large part of the visible light with the wavelength below 520 nm [20,21]. The band gap determines that the maximum theoretical water oxidation photocurrent of BiVO_4_ photoanode is 7.5 mA cm^−2^ under standard solar radiation (AM 1.5 G) and STH efficiency is close to 9.2% [22]. In addition, BiVO_4_ has the advantages of light corrosion resistance. However, the electron mobility of BiVO_4_ is poor [20], which causes an imbalance between the efficiency of light harvest and charge separation in BiVO_4_. This is the bottleneck of BiVO_4_ photoanodes.

It has been reported that the construction of the WO_3_/BiVO_4_ heterojunction can integrate the advantages of WO_3_ and BiVO_4_, where WO_3_ can serve as carrier conduction layer and BiVO_4_ as the main absorber toward visible light [23,24,25]. As a result, the heterojunction can not only widen the range of light utilization, but also promote the effective charge separation [26]. In 2011, Suk Joon Hong et al. first prepared a series of WO_3_/BiVO_4_ heterojunctions with different WO_3_ or BiVO_4_ thicknesses, and it was reported that the obtained photocurrents were closely related to the WO_3_/BiVO_4_ structure [27]. From then, new structures of WO_3_/BiVO_4_ heterojunctions were further developed, such as 1D nanorod core-shell [28,29], 2D nanosheet core-shell [30] and 3D inverse opal structures [31,32]. However, the structural optimization mainly focused on the morphology and pore distribution of the underlying WO_3_; there are still few reports on the upper BiVO_4_ layer. Most studies reported the WO_3_/BiVO_4_ heterojunction adopting the spin or drop coating to deposit a conformal BiVO_4_ layer onto the WO_3_ [33,34,35,36,37,38]. However, this process is complex and needs repeated coating and annealing. Moreover, it often gives a non-uniform coating layer on the surface of WO_3_.

It has been reported that there are mainly three kinds of heterojunction configurations: (1) the planar laminations, which can be easily prepared by combining different semiconductor layers, but the planar structure severely limits the active area of electrodes; (2) the core-shell coaxial structure, which can significantly improve the effective heterojunction area, but the holes generated by the inner semiconductor can only be transmitted to the surface semiconductor through the heterojunction, which might lead to the accumulation of charge carriers at the semiconductor interface; and (3) the surface dispersion structure, which can ensure that both kinds of semiconductor can accept light and participate in the surface reaction, and at the same time remaining a large effective heterojunction area. Therefore, to fabricate the surface dispersed WO_3_/BiVO_4_ heterojunction arrays shows great potential to improve its performance toward PEC water oxidation. Hyungtak Seo et al. [39] used ethyl cellulose as the morphology control agent and prepared BiVO_4_ with island morphology decorated on the WO_3_ layer via the spin coating. Ho Won Jang et al. [40] prepared dot-like BiVO_4_ coated on the entire surface of WO_3_ nanorods by pulse electrodeposition, which could significantly increase the active area of the heterojunction. Kiyoung Lee et al. [41] investigated the interfacial growth of the optimal BiVO_4_ nanoparticles onto WO_3_ nanoplates via a chemical bath deposition method; however, it still could not avoid the ten times of repeated coating procedure. Hongbing Yu et al. [42] synthesized the coral-like WO_3_/BiVO_4_ photoanode via BiOI electrodeposition with a relatively complex process. Huixia Guo et al. [43] reported the synthesis of BiVO_4_ nanoparticles on the WO_3_ thin film by hydrothermal method; however, the structure still needs to be optimized to decrease the particle agglomeration.

In this work, the surface dispersed heterojunction structure of BiVO_4_-nanoparticle@WO_3_-nanoflake was prepared by the hydrothermal combined with solution-thermal method to obtain high PEC water splitting performance. Firstly, WO_3_ nanoflake arrays with optimized properties were obtained by changing the synthesis conditions and controlling the morphology of WO_3_ films by hydrothermal method. Afterwards, uniform BiVO_4_ nanoparticles were covered on the surface of WO_3_ nanoflake arrays through the secondary solvothermal process. As a result of the formation of WO_3_/BiVO_4_ heterojunction and its well-arranged structure, the performance of the surface dispersed WO_3_/BiVO_4_ arrays was significantly improved compared with pure WO_3_ nanoflake arrays.

## 2. Results and Discussion

### 2.1. Structure and Properties of WO_3_ Nanoflake Arrays

**Figure 1:**
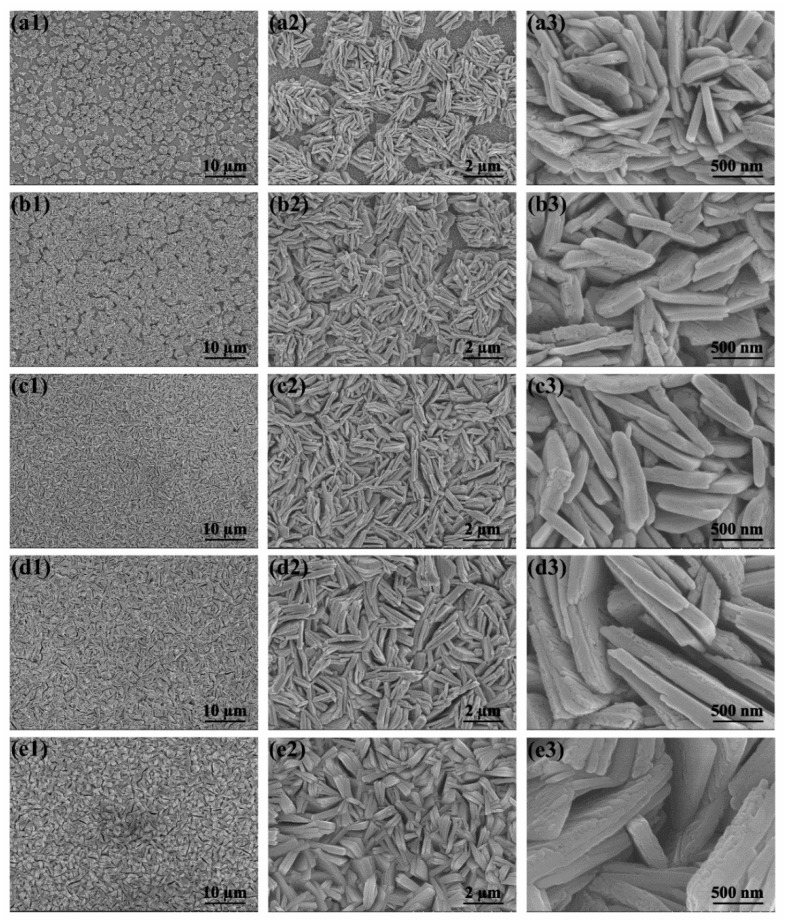
Top view SEM images of different WO_3_ arrays: (**a1**–**a3**) WO_3_-a, (**b1**–**b3**) WO_3_-b, (**c1**–**c3**) WO_3_-c, (**d1**–**d3**) WO_3_-d and (**e1**–**e3**) WO_3_-e.

**Figure 2:**
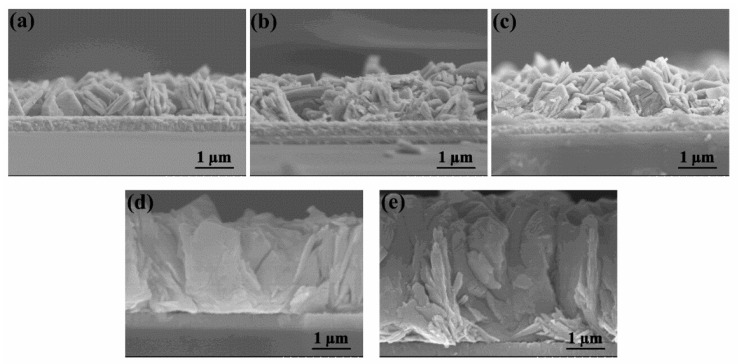
Cross-sectional view SEM images of the WO_3_ arrays: (**a**) WO_3_-a, (**b**) WO_3_-b, (**c**) WO_3_-c, (**d**) WO_3_-d and (**e**) WO_3_-e.

Firstly, the WO_3_ morphology was optimized by changing the concentration of hydrothermal precursors. Figure 1 and Figure 2 exhibit SEM images of different WO_3_ films from the top and cross-sectional view, respectively. It can be seen that all of the WO_3_ films are featured with nanoflake structure, which are arranged directly onto the FTO substrate. When the concentration of sodium tungstate in precursor solution is low (10 mM), nanoflower clusters assembled by thin nanoflakes appear in WO_3_-a, which are uniformly distributed on FTO substrate with a relatively large exposed FTO space. As seen in Figure 2a, the thickness of the WO_3_-a array is about 1.2 μm. As for WO_3_-b, the distribution of the WO_3_ nanoflower structure gets distinctly denser with less exposed area of FTO (Figure 1(b1–b3)) and the thickness of the nanoflake array increases slightly to 1.3 μm (Figure 2b). When the concentration of sodium tungstate solution is increased to 20 mM, no nanoflowers appeared in WO_3_-c, where the morphology in a wide field of vision is the uniform nanoflake array. There is no FTO substrate exposed. As seen in Figure 2c, the thickness of WO_3_-c is about 1.5 μm. When the concentration of sodium tungstate solution is further increased to 50 or 80 mM, the WO_3_-c and WO_3_-d nanoflakes seen from the top view do not change much, with a slightly denser arrangement of the WO_3_ nanoflakes (Figure 1(d1–d3,e1–e3)). However, the thickness of the nanoflake arrays changes significantly.

As seen in Figure 2d, the thickness of WO_3_-d is about 3.2 μm, which is almost twice that of WO_3_-c. When the concentration of the sodium tungstate solution further increases to 80 mM, the thickness of the WO_3_-e array increases to 4.0 μm (Figure 2e). Combined with the images from Figure 1 and Figure 2, it can be concluded that the increase in precursor concentration in the initial stage mainly causes changes in the coverage of the film and the density of the nanoflakes, while the thickness of the WO_3_ array changes little. Whereas, when the nanoflakes completely cover the FTO substrate, further increase in precursor concentration mainly causes the increase in the thickness of the WO_3_ nanoflake array. Moreover, the morphology of nanoflake arrays directly grown on FTO substrates can not only provide a large specific surface area, but also facilitate the efficient transfer of photogenerated electrons to the FTO collector.

**Figure 3:**
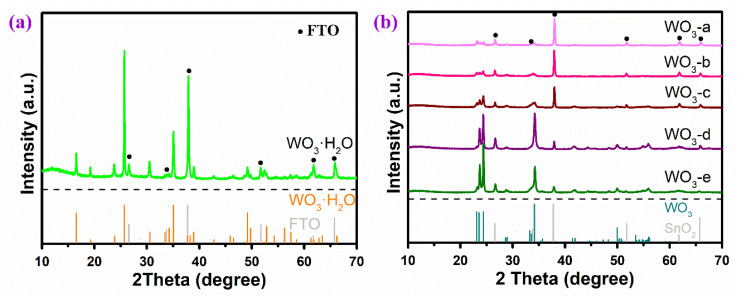
X-ray diffraction (XRD) patterns of WO_3_ arrays (**a**) before and (**b**) after heat treatment.

To confirm the phase composition of the WO_3_ arrays, Figure 3 gives the XRD patterns of WO_3_ arrays obtained through the hydrothermal process before and after heat treatment. Taking WO_3_-d in Figure 3a as an example, before heat treatment, strong diffraction peaks appear at 16.5°, 19.3°, 23.8°, 25.7°, 30.5°, 35.1°, 38.9°, 49.2° and 52.9°, which respectively can be corresponded to the crystal faces of (020), (011), (120), (111), (031), (002), (022), (042) and (222) from WO_3_·H_2_O (JCPDS No. 43-0679). The diffraction peaks marked by black dots are from the FTO substrate. The XRD patterns of WO_3_ arrays prepared after calcination are given in Figure 3b. There are no diffraction peaks corresponding to WO_3_·H_2_O, indicating that all of the WO_3_·H_2_O phase has inversed after heat treatment at 500 °C. Instead, all of the peaks are from monoclinic tungsten oxide (JCPDS 43-1035), where the respective peaks at 23.1°, 23.6°, 24.4°, 34.2° and 50.0° can, respectively, be ascribed to the (002), (020), (200), (202) and (140) crystal planes [44]. In addition, it can be seen that after the same synthesis process, the intensity of WO_3_ diffraction peak gradually increases with the increase in the precursor concentration. These results are consistent with the SEM images that as the concentration of sodium tungstate solution increases, WO_3_ nanoflakes gradually cover the FTO substrate and then increase in thickness.

**Figure 4:**
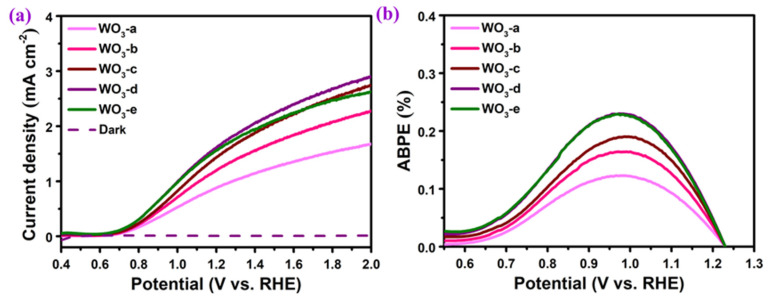
(**a**) Linear sweep voltammograms (LSV) and (**b**) the applied bias photon-to-current efficiency (ABPE) curves of WO_3_ arrays.

The photoelectrochemical water splitting performance of different WO_3_ nanoarrays was studied by using them as the working electrodes and the results are shown in Figure 4. According to the linear sweep voltammograms curve (LSV) in Figure 4a, under illumination the onset potential of each WO_3_ array is similar, which is about 0.69 V (vs. RHE). For WO_3_-a, it has the lowest water splitting performance with a photocurrent at 1.23 V (vs. RHE) being 0.92 mA cm^−2^. According to the UV-Visible light absorption performance (Supplementary Figure S1), WO_3_-a has limited absorption of incident light. The insufficient photogenerated electrons and holes lead to the lowest photocurrent. With the increase in precursor concentration, the photocurrent gradually increases, where the photocurrent of WO_3_-b, WO_3_-c and WO_3_-d is 1.26, 1.50 and 1.69 mA cm^−2^, respectively. When the concentration of precursor solution is further increased, the photocurrent of WO_3_-e decreased to 1.63 mA cm^−2^. Combined with the SEM images, the WO_3_ nanoflake arrays initially get denser and thicker with the increase in the precursor solution concentration, which can increase the light absorption and photocarrier generation so as to improve the photocurrent of photoelectrochemical water oxidation. On the contrary, the thick film of WO_3_-e could extend the distance of photogenerated electrons to the FTO collector, which increases the probability of carrier recombination and is not conducive to the effective separation of carriers. Thus, the WO_3_-d shows the largest photocurrent as a result of a balance of photocarrier generation and separation. Figure 4b shows the curves of applied bias photon-to-current (ABPE) conversion efficiency at the applied bias of 0.98 V (vs. RHE). Among them, the ABPE of WO_3_-d reaches the maximum value at about 0.23%, indicating that it has a good photon-to-current conversion efficiency.

### 2.2. Structure and Properties of WO_3_/BiVO_4_ Nanoarrays

**Figure 5:**
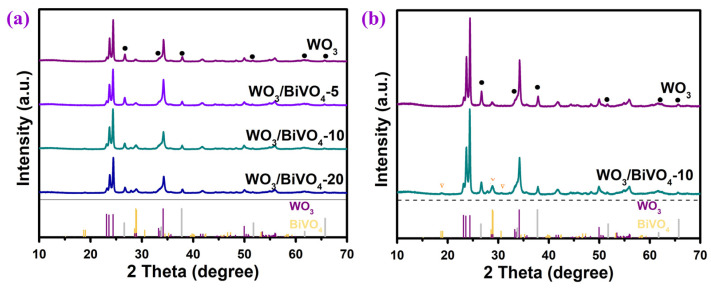
(**a**) X-ray diffraction (XRD) patterns of WO_3_ and WO_3_/BiVO_4_ nanoarrays and (**b**) enlarged X-ray diffraction (XRD) patterns of WO_3_ and WO_3_/BiVO_4_-10.

Based on the structure and properties of the WO_3_ arrays, WO_3_-d was chosen to composite BiVO_4_ for further research. Figure 5 gives the XRD patterns of the WO_3_/BiVO_4_ composite array prepared with different concentrations of BiVO_4_ precursors. In addition to the peaks of WO_3_ and FTO substrate (marked with black circles), new weak peaks appear, and the intensity of these peaks gradually increases with the increase in the concentration of BiVO_4_ precursor solution. Taking WO_3_ and WO_3_/BiVO_4_-10 as an example, it can be seen from the magnified XRD in Figure 5b that new diffraction peaks at 18.7°, 28.6°, 28.8°, 28.9° and 30.5° in WO_3_/BiVO_4_-10 can, respectively, be corresponded to the (110), (−130), (−121), (121) and (040) crystal faces of monoclinic scheelite BiVO_4_ (JCPDS No. 14-0688) [45]. Therefore, the existence of BiVO_4_ is proved, and the relatively weak diffraction peaks of BiVO_4_ might be ascribed to the low content of BiVO_4_. The small peak around 27.8° can be assigned to the (011) crystal face of the tiny VO_2_ (JCPDS No. 43-1051) after the hydrothermal route.

**Figure 6:**
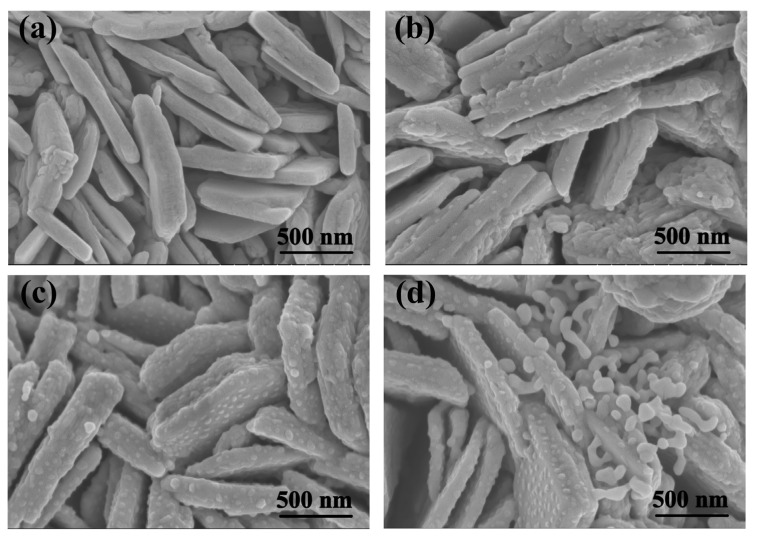
SEM images of WO_3_ and WO_3_/BiVO_4_ composite arrays; (**a**) WO_3_, (**b**) WO_3_/BiVO_4_-5, (**c**) WO_3_/BiVO_4_-10 and (**d**) WO_3_/BiVO_4_-20.

The morphology of the WO_3_/BiVO_4_ composite prepared with different concentrations of BiVO_4_ precursor solution were observed by SEM. As shown in Figure 6, the nanoflake structure of the WO_3_ array was still maintained after solvothermal reaction. From the morphology of WO_3_/BiVO_4_-5 in Figure 6b, small nanoparticles can be clearly verified on the WO_3_ nanoflake with a particle size of ~20 nm, which are uniformly attached to the WO_3_ nanoflakes. As the reaction concentration of precursors increases, the distributed BiVO_4_ on the surface of the WO_3_ nanoflake becomes denser. For WO_3_/BiVO_4_-10, it can be seen from Figure 6c that the size of BiVO_4_ nanoparticles increases to ~50 nm and there is a uniform dispersion of BiVO_4_ nanoparticles on the surface of the entire WO_3_ nanoflake array. When the reaction concentration further rises to 20 mM, as shown in Figure 6d, WO_3_/BiVO_4_-20 is covered with not only uniform BiVO_4_ nanoparticles but also aggregated nanoparticles. As shown in Supplementary Figure S2, the size of large BiVO_4_ particles in WO_3_/BiVO_4_-20 can be as large as ~1 μm. Therefore, by controlling the proper precursor concentration, the surface dispersed morphology of BiVO_4_ nanoparticles on the surface of WO_3_ nanoflakes is fabricated, which is conducive to expanding the interfacial surface area and increasing the reactive sites.

The cross-sectional SEM image of WO_3_/BiVO_4_-10 is illustrated in Supplementary Figure S3. The thickness of WO_3_/BiVO_4_-10 is similar to that of the pure WO_3_ arrays, which means the solvothermal progress does not change the morphology of WO_3_ arrays. BiVO_4_ nanoparticles can also be verified from the cross-sectional view, further indicating the formation of the surface dispersed morphology of BiVO_4_-nanoparticle@WO_3_-nanoflake. Sn distribution is consistent with the position of FTO substrate. From the element mapping, Bi, V, W and O elements are distributed uniformly, indicating that BiVO_4_ particles are uniformly loaded on the entire longitudinal WO_3_ nanoflakes.

**Figure 7:**
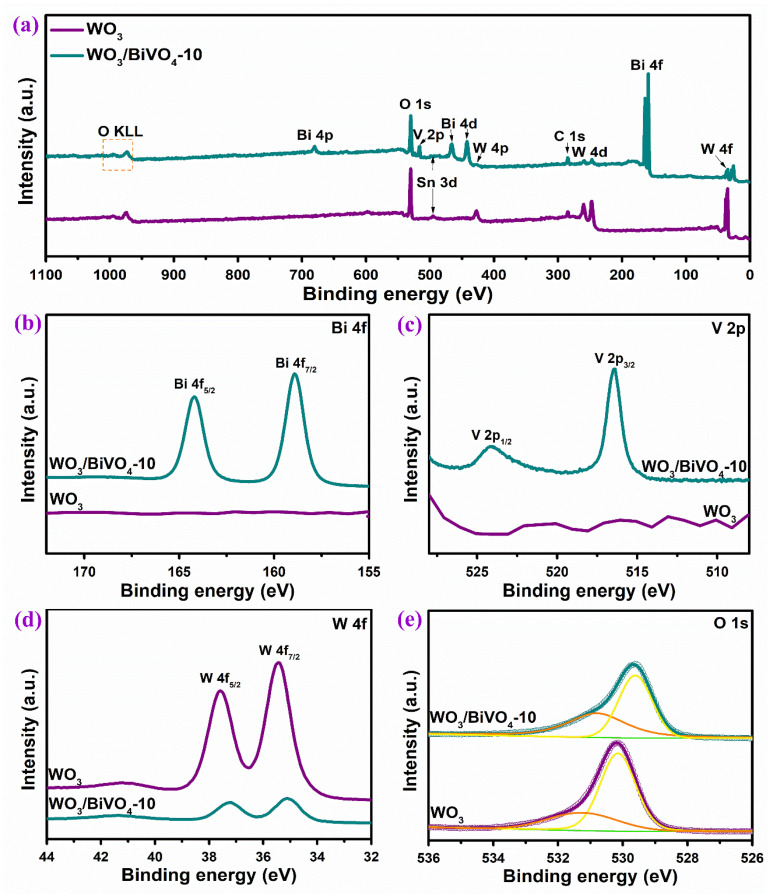
XPS spectra of WO_3_ and WO_3_/BiVO_4_-10, (**a**) survey spectrum where the orange square and the black arrows represent the binding energy of the corresponding element. High-resolution XPS spectra of (**b**) Bi 4p, (**c**) V 2p, (**d**) W 4f and (**e**) O 1s.

Representative XPS spectra were tested to study the chemical elemental composition and the corresponding elemental valence states of the WO_3_ and WO_3_/BiVO_4_ arrays. The survey spectrum in Figure 7a proves that WO_3_ contains W and O elements, while WO_3_/BiVO_4_-10 contains Bi, V, W and O elements. From the high-resolution spectra of Bi 4f and V 2p in Figure 7b,c, the WO_3_ array shows basically a straight line. After BiVO_4_ loaded, WO_3_/BiVO_4_-10 showed two peaks at 164.2 and 158.9 eV, ascribed to the binding energy of Bi 4f_5/2_ and Bi 4f_7/2_, respectively. This indicates that the Bi element is presented in the form of Bi^3+^ [46]. The binding energies at 524.1 and 516.5 eV indicate the existence of V 2p_1/2_ and V 2p_3/2_ [47]. According to the high-resolution XPS spectra of W 4f in Figure 7d, the binding energies of W 4f_5/2_ and W 4f_7/2_ in the WO_3_ nanoarrays are located at 35.4 and 37.6 eV, which proves the chemical state of W element in the form of W^6+^ [44]. For the WO_3_/BiVO_4_ composite array, the intensity of W 4f spectrum is obviously weakened after the deposition of BiVO_4_ on WO_3_. In addition, the binding energy of W 4f moves towards the lower energy direction, which proves that there is a strong interaction between WO_3_ and BiVO_4_, which is beneficial for electron conduction. The high-resolution XPS spectra of O 1s shown in Figure 7e can be fitted into two peaks located at 530.1 and 531.4 eV, corresponding to lattice oxygen and defect sites with low oxygen coordination (i.e., hydroxyl), respectively.

**Figure 8:**
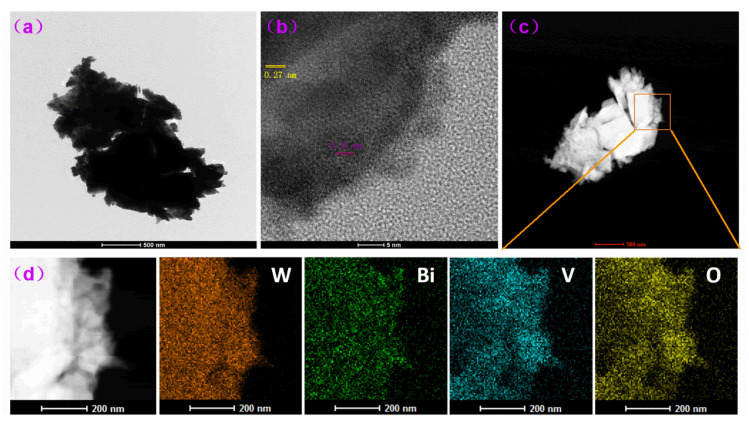
(**a**) TEM, (**b**) HRTEM and (**c**,**d**) EDS element distribution images of WO_3_/BiVO_4_-10 arrays ((**d**) zooms in of marked square region in (**c**)).

The WO_3_/BiVO_4_-10 composite array was scraped off the substrate for TEM test. A representative image is shown in Figure 8a and its corresponding HRTEM image is shown in Figure 8b, where the lattice spacing from the nanoflake structure is around 0.27 nm, ascribed to the (022) interplanar spacing of monoclinic WO_3_. The lattice spacing of surface loaded particles is ~0.31 nm, which is consistent with the (−121) crystal plane of BiVO_4_. In addition, the tight lattice connection between BiVO_4_ and WO_3_ further indicates a good binding between BiVO_4_ and WO_3_. From Figure 8c, the nanoflake structure of WO_3_ can be distinguished. As shown in the element distribution images (Figure 8d), W element can be seen in the whole field of vision, while Bi and V elements are relatively distributed in the marginal region corresponding to small particles. The distribution of Bi, V, W and O elements indicates that BiVO_4_ is evenly distributed on the surface of WO_3_.

**Figure 9:**
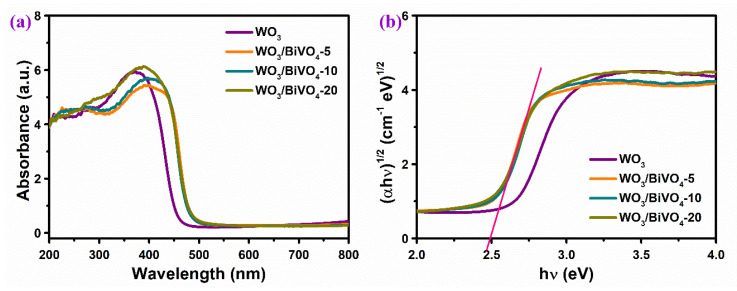
(**a**) UV-vis absorption spectra and (**b**) Tauc plots of WO_3_ and WO_3_/BiVO_4_ arrays.

Figure 9 is the optical absorption performance of WO_3_ and WO_3_/BiVO_4_ arrays. Pure WO_3_ exhibits a light response to ultraviolet light and part of visible light. After loading BiVO_4_, the light response range of the WO_3_/BiVO_4_ array shows an obvious red shift, but the light absorption intensity is similar in the ultraviolet region. The results show that the absorbed light of 400–480 nm is mainly due to the contribution of BiVO_4_, and the utilization of visible light by WO_3_/BiVO_4_ is significantly improved after loading BiVO_4_. The corresponding Tauc curve is given in Figure 9b, where the band gap can be estimated by extending the linear part of the curve. The band gap of the WO_3_/BiVO_4_ composite array is about 2.50 eV, which is significantly reduced compared with that of the pure WO_3_ array (2.64 eV), indicating a wider light spectrum of the WO_3_/BiVO_4_ composite.

**Figure 10:**
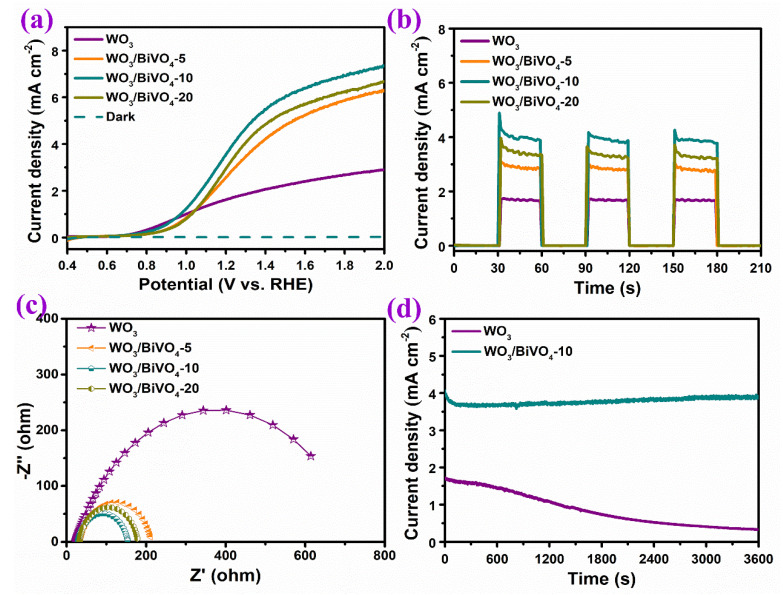
(**a**) Linear sweep voltammograms (LSV), (**b**) amperometric i–t curves under chopped light illumination, (**c**) electrochemical impedance spectra (EIS) and (**d**) the stability of WO_3_ and WO_3_/BiVO_4_ arrays.

The performance of WO_3_ and WO_3_/BiVO_4_ toward photoelectrochemical water oxidation was measured by using the WO_3_ and WO_3_/BiVO_4_ arrays as photoanodes. As shown in the linear sweep voltammograms curves (Figure 10a), the properties of WO_3_/BiVO_4_ are significantly improved compared with pure WO_3_. The dashed line represents the LSV curve of WO_3_/BiVO_4_ in the dark, which indicates the significant photoresponse of all photoanodes. As mentioned above, the photocurrent at 1.23 V (vs. RHE) of pure WO_3_ is 1.69 mA cm^−2^. After loading BiVO_4_, the photocurrent of WO_3_/BiVO_4_-5 was significantly increased to 2.57 mA cm^−2^. According to the above analysis, the absorption of visible light in the WO_3_/BiVO_4_ composite array is significantly increased, and the formation of the heterojunction structure between WO_3_ and BiVO_4_ is conducive to the effective separation of photogenerated carriers. As for the WO_3_/BiVO_4_-10 photoanode, the highest photocurrent at 3.53 mA cm^−2^ is observed. This performance may be due to the appropriate loading amount and excellent dispersion of BiVO_4_ in WO_3_/BiVO_4_-10, which enables the sufficient light absorption and effective carrier separation at the same time. As a result, more carriers can effectively transfer to the electrode surface for PEC OER reactions to take place. The key characteristics of recent reported WO_3_/BiVO_4_ heterojunction photoanodes without an additional catalyst or doping for solar water oxidation are summarized in Table 1. Considering that the photocurrent can be significantly improved if there is the hole scavenge in the electrolyte, the performance of the surface dispersed WO_3_/BiVO_4_ nanoplates in our work are among the best. In the case of WO_3_/BiVO_4_-20, the photocurrent then decreases to 2.95 mA cm^−2^. It has been reported that the short life of photogenerated electrons is the main drawback of the PEC performance of BiVO_4_. The agglomeration and the existence of large size BiVO_4_ in WO_3_/BiVO_4_-20 might hinder the effective carrier transfer, resulting in the decreased photocurrent in spite of the increased BiVO_4_ loading. Figure 10b shows the i–t curves of WO_3_ and the WO_3_/BiVO_4_ arrays measured with chopped light illumination. All the photoelectrodes exhibit prompt response toward light, indicating that the photoanode material has excellent transfer ability of photogenerated electrons and holes. The photocurrents of WO_3_, WO_3_/BiVO_4_-5, WO_3_/BiVO_4_-10 and WO_3_/BiVO_4_-20 photoanodes are 1.69, 2.84, 3.92 and 2.30 mA cm^−2^, which is consistent with the trend of LSV results.

The electrochemical impedance spectra of WO_3_ and WO_3_/BiVO_4_ arrays are shown in Figure 10c to characterize the impedance at the semiconductor/electrolyte interface. The corresponding equivalent circuit is provided in Supplementary Figure S4. R_S_ stands for the series resistance. CPE is constant phase element and R_ct_ represents the charge transfer resistance of the electrode/electrolyte interface. The radius of the arc in the impedance spectrum indicates the charge transfer ability of the photoanodes, where the smaller the radius means the smaller the impedance and a better charge transfer ability. Compared with pure WO_3_, the charge transfer impedance of WO_3_/BiVO_4_ decreases significantly, indicating that compositing BiVO_4_ can effectively reduce the charge transfer impedance of the photoelectrode due to the formation of the heterojunction. Among the WO_3_/BiVO_4_ composite arrays, WO_3_/BiVO_4_-10 has the lowest charge transfer impedance, which is the most favorable for photogenerated carriers to transfer to the electrode surface to oxidize water. The arc radius of WO_3_/BiVO_4_-20 is larger than that of WO_3_/BiVO_4_-10, which further indicates that the larger charge transfer impedance of WO_3_/BiVO_4_-20 limits its PEC water oxidation performance. This is consistent with the morphology analysis results. Figure 10d is the photocurrent stability test of WO_3_ and WO_3_/BiVO_4_-10 measured at 1.23 V (vs. RHE). Since WO_3_ is easily oxidized, the stability is poor. After loading BiVO_4_, the stability of WO_3_/BiVO_4_-10 was significantly improved; there was no significant drop during subsequent tests except for the initial current drop. The protective effect of the BiVO_4_ layer for WO_3_ can be verified. The formation of the WO_3_/BiVO_4_ heterojunction contributes to the enhanced separation of charge carriers and relieves the charge accumulation on the WO_3_ surface.

**Figure 11:**
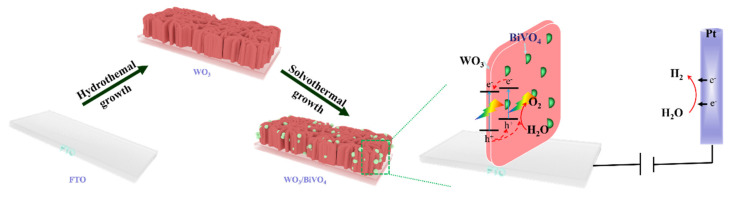
Schematic illustration of the formation and the PEC water oxidation mechanism of the WO_3_/BiVO_4_ arrays. The red flakes represent WO_3_ and the green particles represent BiVO_4_.

The fabrication and the PEC water oxidation mechanism of the surface dispersed WO_3_/BiVO_4_ heterojunction is schematically illustrated in Figure 11. The BiVO_4_-nanoparticle@ WO_3_-nanoflake structure, verified by the SEM and TEM measurements, can be prepared by carefully controlling the hydrothermal and solvothermal conditions. When used as photoanodes, both WO_3_ and BiVO_4_ can absorb light and generate an enhanced charge carrier. Subsequently, the WO_3_/BiVO_4_ heterojunction promotes the directional migration of photogenerated carriers and, thus, improves charge separation across the WO_3_ and BiVO_4_ interface, where the WO_3_ layer with better charge transfer ability has proper thickness to work as a skeleton to optimize the light absorption and provide the electron pathway to the FTO substrate at the same time. Meanwhile, the BiVO_4_ nanoparticles with little size promote light absorption and facilitate charge carrier transfer. In addition, the surface dispersed structure of the BiVO_4_-nanoparticle@ WO_3_-nanoflake ensures that the photogenerated holes from both WO_3_ and BiVO_4_ can contribute to PEC water oxidation, which greatly helps to suppress the accumulation and recombination of charge carriers at the semiconductor interface, as confirmed by the increased photocurrent as well as stability.

## 3. Materials and Methods

### 3.1. Preparation of the WO_3_ Nanoflake Arrays

All chemical reagents used in this work are analytical reagent. The tungsten trioxide nanoflake arrays were grown directly on the surface of F doped tin oxide (FTO) glass by the hydrothermal method, with sodium tungstate as tungsten source and oxalic acid as morphology control agent. Specifically, a certain amount of sodium tungstate and oxalic acid was weighed and dissolved completely in deionized water, where the molar ratio of Na_2_WO_4_ and H_2_C_2_O_4_ was controlled at 1:2. Then, 0.5 mL of concentrated HCl was dropwise added into the above mixed solution. A piece of FTO conductive glass was placed in a reaction kettle and a certain amount of the above precursor solution was added. After the hydrothermal treatment at 150 °C for 4 h, we can observe a yellow film on the surface of the FTO. Finally, the WO_3_ nanoflake arrays were obtained by heat treatment at 500 °C for 2 h in air. In order to optimize the synthesis conditions of the WO_3_ film, precursors with different concentrations of sodium tungstate of 10, 15, 20, 50 and 80 mM were prepared and the corresponding WO_3_ films were denoted as WO_3_-a, WO_3_-b, WO_3_-c, WO_3_-d and WO_3_-e.

### 3.2. Preparation of the WO_3_/BiVO_4_ Nanoflake Arrays

The WO_3_/BiVO_4_ composite films were further prepared by the solvothermal method. A certain amount of Bi(NO_3_)_3_·5H_2_O was weighed and dissolved in ethylene glycol to obtain a colorless transparent solution. Afterwards, the equal molar mass NH_4_VO_3_ was added and stirred at an accelerated speed for another 20 min to form a uniform yellow suspension. The above-obtained WO_3_ film on FTO was placed in a reaction kettle. After the solvothermal reaction at 160 °C for 4 h, the film was fully rinsed with distilled water, dried in the oven at 60 °C and changed into the WO_3_/BiVO_4_ composite film by heat treatment at 500 °C in air. By controlling the concentration of Bi(NO_3_)_3_·5H_2_O and NH_4_VO_3_ solution at 5, 10 and 20 mM, the WO_3_/BiVO_4_ films with different amounts of BiVO_4_ were prepared, which were abbreviated as WO_3_/BiVO_4_-5, WO_3_/BiVO_4_-10 and WO_3_/BiVO_4_-20, respectively.

### 3.3. Characterization

The X-ray diffraction (XRD) data of all samples were collected on Bruker’s D8 advance diffractometer (Karlsruhe, Germany). The surface chemical composition of sample was analyzed by the X-ray photoelectron spectra (XPS) on an ESCALab220i-XL electron spectrometer (Thermo Scientific, Waltham, MA, USA). The Hitachi S4800 (Tokyo, Japan) was used to obtain the scanning electron microscope (SEM) images. The transmission electron microscopy (TEM) images and the elemental mapping images were measured on Philips Tecnai G2 (Thermo Fisher, Waltham, MA, USA). UV-vis diffuse reflectance spectra were reflected by the UV-3600 spectrophotometer (Shimadzu, Tokyo, Japan).

### 3.4. Photoelectrochemical Tests

The photoelectrochemical tests of the photoanodes were measured by the three-electrode system on photoelectrochemical workstation (Zahner IM6, Zahner, Kronach, Germany), where the photoanode was used as the working electrode, Pt electrode as the counter electrode and saturated calomel electrode as the reference electrode. The simulated standard sunlight was the light source. The 0.2 mol L^−1^ potassium phosphate buffer solution (pH = ~7) was used as electrolyte. PEC water oxidation was evaluated by the linear sweep voltammograms (LSV). The electrochemical impedance spectra (EIS) were measured from a frequency of 100,000 Hz to 0.1 Hz with a disturbing current voltage of 10 mV.

## 4. Conclusions

The WO_3_/BiVO_4_ composite photoanode with a structure of surface dispersed heterojunction was constructed, where a monoclinic WO_3_ nanoflake array was grown on FTO substrate, with optimized morphology and thickness by controlling the appropriate concentration of precursor solution. The BiVO_4_ nanoparticles were then uniformly loaded on the WO_3_ nanoflakes using the solvothermal method. When used as photoanode, the WO_3_/BiVO_4_ composite exhibits superior PEC water oxidation performance with photocurrent of 3.53 mA cm^−2^, which is twice that of pure WO_3_ photoanode. This improved performance comes from the advantages of the unique surface dispersed WO_3_/BiVO_4_ array structure, as follows: (1) the proper thickness of the WO_3_/BiVO_4_ composites balanced the light absorption and the charge separation, contributing to more charge carriers reaching the interface; (2) the formation of the heterojunction immensely improves the separation efficiency of interfacial charge carriers; and (3) the surface dispersed heterojunction structure of BiVO_4_-nanoparticle@WO_3_-nanoflake provides a large heterojunction interface, which offers a large surface area and improves the stability of the photoelectrode.

## Figures and Tables

**Table 1:** Recent reports on the WO_3_/BiVO_4_ heterojunction photoanodes for solar water oxidation.

Structure of Photoelectrodes	Method	Electrolyte	Photocurrent Density at 1.23 V vs. RHE (mA cm^−2^)	Ref.
BiVO_4_ layer/nanoplate-like WO_3_	hydrothermal method + spin coating (20 cycles)	0.5 M Na_2_SO_4_	0.43	[34]
BiVO_4_ layer/WO_3_ nanoplates	hydrothermal method + spin coating	0.5 M Na_2_SO_4_	0.62	[35]
BiVO_4_ layers/a WO_3_ layer	spin-coating + repeated spin-coating	0.5 M Na_2_SO_4_	1.25	[36]
BiVO_4_ layer/WO_3_ nanoplates	hydrothermal method + spin-coating deposition (4 cycles)	0.5 M Na_2_SO_4_	1.93	[37]
WO_3_ nanoplates coated by BiVO_4_ nanoparticles	hydrothermal + spinning calcination	0.1 M KPi buffer solution	1.2	[38]
Island-like BiVO_4_/Plate-like WO_3_	hydrothermal + spin coating	0.1 M Na_2_SO_4_	4.2	[39]
BiVO_4_ nanodot/WO_3_ nanorods	glancing angle deposition + Pulsed electrodeposition	0.5 M KPi + 1 M Na_2_SO_3_	4.55	[40]
BiVO_4_ nanoparticles/WO_3_ nanoplates	hydrothermal method + chemical bath deposition (20 times)	0.5 M Na_2_SO_4_	1.7	[41]
coral-like BiVO_4_/WO_3_	hydrothermal method + electrodeposition process	0.5 M phosphate buffer solution	2.9	[42]
BiVO_4_ nanoparticles/WO_3_ nanoplates	hydrothermal method + hydrothermal method	0.5 M (Na_2_SO_4_ + Na_2_SO_3_)	1.35	[43]
surface dispersed BiVO_4_/WO_3_ nanoplates	hydrothermal method + solvothermal method	0.2 M KPi buffer solution	3.53	This work

## Data Availability

Data are contained within the article and Supplementary Materials.

## Supplementary Materials

The following supporting information can be downloaded at: https://www.mdpi.com/article/10.3390/molecules29020372/s1, Figure S1: (a) UV-vis absorption spectra and (b) Tauc plots of WO_3_ arrays; Figure S2: SEM images of WO_3_/BiVO_4_ composite arrays, (a) WO_3_/BiVO_4_-5, (b) WO_3_/BiVO_4_-10, (c) WO_3_/BiVO_4_-20; Figure S3: Cross-sectional view SEM image of WO_3_/BiVO_4_-10 and the corresponding elemental mapping; Figure S4: The equivalent circuit of the electrochemical impedance spectra.

## References

[1] Samuel E., Joshi B., Kim M.W., Swihart M.T., Yoon S.S. (2020). Morphology engineering of photoelectrodes for efficient photoelectrochemical water splitting. Nano Energy.

[2] Mushtaq M.A., Kumar A., Yasin G., Arif M., Tabish M., Ibraheem S., Cai X., Ye W., Fang X., Saad A. (2022). 3D interconnected porous Mo-doped WO_3_@CdS hierarchical hollow heterostructures for efficient photoelectrochemical nitrogen reduction to ammonia. Appl. Catal. B Environ..

[3] Daulbayev C., Sultanov F., Bakbolat B., Daulbayev O. (2020). 0D, 1D and 2D nanomaterials for visible photoelectrochemical water splitting. A Review. Int. J. Hydrogen Energy.

[4] Marwat M.A., Humayun M., Afridi M.W., Zhang H., Karim M.R.A., Ashtar M., Usman M., Waqar S., Ullah H., Wang C. (2021). Advanced catalysts for photoelectrochemical water splitting. ACS Appl. Energy Mater..

[5] Tang J., Durrant J.R., Klug D.R. (2008). Mechanism of photocatalytic water splitting in TiO_2_. Reaction of water with photoholes, importance of charge carrier dynamics, and evidence for four-hole chemistry. J. Am. Chem. Soc..

[6] Yao B., Zhang J., Fan X., He J., Li Y. (2019). Surface engineering of nanomaterials for photo-electrochemical water splitting. Small.

[7] Zhang X., Wang X., Wang D., Ye J. (2019). Conformal BiVO_4_-layer/WO_3_-nanoplate-array heterojunction photoanode modified with cobalt phosphate cocatalyst for significantly enhanced photoelectrochemical performances. ACS Appl. Mater. Interfaces.

[8] Li H., Lin C., Yang Y., Dong C., Min Y., Shi X., Wang L., Lu S., Zhang K. (2023). Boosting Reactive Oxygen Species Generation Using Inter-Facet Edge Rich WO_3_ Arrays for Photoelectrochemical Conversion. Angew. Chem. Int. Ed..

[9] Han H., Riboni F., Karlický F., Kment S., Goswami A., Sudhagar P., Yoo J., Wang L., Tomanec O., Petr M. (2017). α-Fe_2_O_3_/TiO_2_ 3D hierarchical nanostructures for enhanced photoelectrochemical water splitting. Nanoscale.

[10] Wang L., Si W., Ye Y., Wang S., Hou F., Hou X., Cai H., Dou S.X., Liang J. (2021). Cu-ion-implanted and polymeric carbon nitride-decorated TiO_2_ nanotube array for unassisted photoelectrochemical water splitting. ACS Appl. Mater. Interfaces.

[11] Chaulagain N., Alam K.M., Kadian S., Kumar N., Garcia J., Manik G., Shankar K. (2022). Synergistic enhancement of the photoelectrochemical performance of TiO_2_ nanorod arrays through embedded plasmon and surface carbon nitride co-sensitization. ACS Appl. Mater. Interfaces.

[12] Han J., Liu Z. (2021). Optimization and modulation strategies of zinc oxide-based photoanodes for highly efficient photoelectrochemical water splitting. ACS Appl. Energy Mater..

[13] Xie X., Wang R., Ma Y., Chen J., Cui Q., Shi Z., Li Z., Xu C. (2022). Photothermal-effect-enhanced photoelectrochemical water splitting in MXene-nanosheet-modified ZnO nanorod arrays. ACS Appl. Nano Mater..

[14] Zhang Y., Zhang H., Ji H., Ma W., Chen C., Zhao J. (2016). Pivotal role and regulation of proton transfer in water oxidation on hematite photoanodes. J. Am. Chem. Soc..

[15] Wang P., Wang S., Gao L., Long X., Chai H., Li F., Wang Q., Jin J. (2022). Achieving surface-sealing of hematite nanoarray photoanode with controllable metal–organic frameworks shell for enhanced photoelectrochemical water oxidation. J. Catal..

[16] Yuan S.Y., Jiang L.W., Hu J.S., Liu H., Wang J.J. (2023). Fully dispersed irox atomic clusters enable record photoelectrochemical water oxidation of hematite in acidic media. Nano Lett..

[17] Kong W., Zhang X., Liu S., Zhou Y., Chang B., Zhang S., Fan H., Yang B. (2019). N doped carbon dot modified WO_3_ nanoflakes for efficient photoelectrochemical water oxidation. Adv. Mater. Interfaces.

[18] Wu H., Liu Q., Zhang L., Tang Y.W., Wang G., Mao G.B. (2021). Novel nanostructured WO_3_@prussian blue heterojunction photoanodes for efficient photoelectrochemical water splitting. ACS Appl. Energy Mater..

[19] Bu Y., Ren J., Zhang H., Yang D., Chen Z., Ao J.P. (2018). Photogenerated-carrier separation along edge dislocation of WO_3_ single-crystal nanoflower photoanode. J. Mater. Chem. A.

[20] Kim J.H., Lee J.S. (2019). Elaborately modified BiVO_4_ photoanodes for solar water splitting. Adv. Mater..

[21] Lin J., Han X., Liu S., Lv Y., Li X., Zhao Y., Li Y., Wang L., Zhu S. (2023). Nitrogen-doped cobalt-iron oxide cocatalyst boosting photoelectrochemical water splitting of BiVO_4_ photoanodes. Appl. Catal. B Environ..

[22] Jiang W., An Y., Wang Z., Wang M., Bao X., Zheng L., Cheng H., Wang P., Liu Y., Zheng Z. (2022). Stress-induced BiVO_4_ photoanode for enhanced photoelectrochemical performance. Appl. Catal. B Environ..

[23] Grigioni I., Stamplecoskie K.G., Selli E., Kamat P.V. (2015). Dynamics of photogenerated charge carriers in WO_3_/BiVO_4_ heterojunction photoanodes. J. Phys. Chem. C.

[24] Afroz K., Moniruddin M., Bakranov N., Kudaibergenov S., Nuraje N. (2018). A heterojunction strategy to improve the visible light sensitive water splitting performance of photocatalytic materials. J. Mater. Chem. A.

[25] Tateno H., Chen S.Y., Miseki Y., Nakajima T., Mochizuki T., Sayama K. (2022). Photoelectrochemical oxidation of glycerol to dihydroxyacetone over an acid-resistant Ta: BiVO_4_ photoanode. ACS Sustain. Chem. Eng..

[26] Kim J.-H., Kim D.H., Yoon J.W., Dai Z., Lee J.-H. (2019). Rational design of branched WO_3_ nanorods decorated with BiVO_4_ nanoparticles by all-solution processing for efficient photoelectrochemical water splitting. ACS Appl. Energy Mater..

[27] Hong S.J., Lee S., Jang J.S., Lee J.S. (2011). Heterojunction BiVO_4_/WO_3_ electrodes for enhanced photoactivity of water oxidation. Energy Environ. Sci..

[28] Rao P.M., Cai L., Liu C., Cho I.S., Lee C.H., Weisse J.M., Yang P., Zheng X. (2014). Simultaneously efficient light absorption and charge separation in WO_3_/BiVO_4_ core/shell nanowire photoanode for photoelectrochemical water oxidation. Nano Lett..

[29] Shi X., Choi Y., Zhang K., Kwon J., Kim D.Y., Lee J.K., Oh S.H., Kim J.K., Park J.H. (2014). Efficient photoelectrochemical hydrogen production from bismuth vanadate-decorated tungsten trioxide helix nanostructures. Nat. Commun..

[30] Ma Z., Song K., Wang L., Gao F., Tang B., Hou H., Yang W. (2019). WO_3_/BiVO_4_ type-II heterojunction arrays decorated with oxygen-deficient ZnO passivation layer: A highly efficient and stable photoanode. ACS Appl. Mater. Interfaces.

[31] Ma M., Kim J.K., Zhang K., Shi X., Kim S.J., Moon J.H., Park J.H. (2014). Double-deck inverse opal photoanodes: Efficient light Absorption and charge separation in heterojunction. Chem. Mater..

[32] Ma M., Shi X., Zhang K., Kwon S., Li P., Kim J.K., Phu T.T., Yi G.-R., Park J.H. (2016). A 3D triple-deck photoanode with a strengthened structure integrality: Enhanced photoelectrochemical water oxidation. Nanoscale.

[33] Zeng Q., Li J., Li L., Bai J., Xia L., Zhou B. (2017). Synthesis of WO_3_/BiVO_4_ photoanode using a reaction of bismuth nitrate with peroxovanadate on WO_3_ film for efficient photoelectrocatalytic water splitting and organic pollutant degradation. Appl. Catal. B Environ..

[34] Park E., Patil S.S., Lee H., Kumbhar V.S., Lee K. (2021). Photoelectrochemical H_2_ evolution on WO_3_/BiVO_4_ enabled by single-crystalline TiO2 overlayer modulations. Nanoscale.

[35] Patil S.S., Lee J., Park E., Nagappagari L.R., Lee K. (2021). Interstitial M^+^ (M^+^ = Li^+^ or Sn^4+^) doping at interfacial BiVO_4_/WO_3_ to promote photoelectrochemical hydrogen production. ACS Appl. Energy Mater..

[36] Grigioni I., Liberto G.D., Dozzi M.V., Tosoni S., Pacchioni G., Selli E. (2021). WO_3_/BiVO_4_ photoanodes: Facets matching at the heterojunction and BiVO_4_ layer thickness effects. ACS Appl. Energy Mater..

[37] Quang N.D., Van P.C., Majumder S., Jeong J.-R., Kim D., Kim C. (2022). Optimization of photogenerated charge transport using type-II heterojunction structure of CoP/BiVO_4_:WO_3_ for high efficient solar-driver water splitting. J. Alloys Compd..

[38] Li Y., Mei Q., Liu Z., Hu X., Zhou Z., Huang J., Bai B., Liu H., Ding F., Wang Q. (2022). Fluorine-doped iron oxyhydroxide cocatalyst: Promotion on the WO_3_ photoanode conducted photoelectrochemical water splitting. Appl. Catal. B Environ..

[39] Kalanur S.S., Yoo I.-H., Park J., Seo H. (2017). Insights into the electronic bands of WO_3_/BiVO_4_/TiO_2_, revealing high solar water splitting efficiency. J. Mater. Chem. A.

[40] Lee M.G., Kim D.H., Sohn W., Moon C.W., Park H., Lee S., Jang H.W. (2016). Conformally coated BiVO_4_ nanodots on porosity-controlled WO_3_ nanorods as highly efficient type II heterojunction photoanodes for water oxidation. Nano Energy.

[41] Kumbhar V.S., Lee H., Lee J., Lee K. (2019). Interfacial growth of the optimal BiVO_4_ nanoparticles onto self-assembled WO_3_ nanoplates for efficient photoelectrochemical water splitting. J. Colloid Interface Sci..

[42] Chi Z., Zhao J., Zhang Y., Yu H., Yu H. (2022). Coral-like WO_3_/BiVO_4_ photoanode constructed via morphology and facet engineering for antibiotic wastewater detoxifcation and hydrogen recovery. Chem. Eng. J..

[43] Guo H., Zhang Y., Wang S., Li L., Wang W., Sun Q. (2022). In-situ generation of Bi_2_S_3_ to construct WO_3_/BiVO_4_/Bi_2_S_3_ heterojunction for photocathodic protection of 304SS. J. Electroanal. Chem..

[44] Li L., Xiao S., Li R., Cao Y., Chen Y., Li Z., Li G., Li H. (2018). Nanotube array-like WO_3_ photoanode with dual-layer oxygen evolution cocatalysts for photoelectrocatalytic overall water splitting. ACS Appl. Energy Mater..

[45] Baek J.H., Kim B.J., Han G.S., Hwang S.W., Kim D.R., Cho I.S., Jung H.S. (2017). BiVO_4_/WO_3_/SnO_2_ double-heterojunction photoanode with enhanced charge separation and visible-transparency for bias-free solar water-splitting with a perovskite solar cell. ACS Appl. Mater. Interfaces.

[46] Gao Y., Li Y., Yang G., Li S., Xiao N., Xu B., Liu S., Qiu P., Hao S., Ge L. (2018). Fe_2_TiO_5_ as an efficient co-catalyst to improve the photoelectrochemical water splitting performance of BiVO_4_. ACS Appl. Mater. Interfaces.

[47] Chen H.-Q., Lin L.-Y., Chen S.-L. (2018). Direct growth of BiVO_4_/Bi_2_S_3_ nanorod array on conductive glass as photocatalyst for enhancing the photoelectrochemical performance. ACS Appl. Energy Mater..

