# Supplementary material for: Preparation of Surface Dispersed WO3/BiVO4 Heterojunction Arrays and Their Photoelectrochemical Performance for Water Splitting
Source: Molecules. 2024 Jan 11;29(2):372. doi: 10.3390/molecules29020372 (PMC10818345; doi:10.3390/molecules29020372)
Supplement: Supplementary file 1 [file molecules-29-00372-s001.zip › molecules-2741719-supplementary.pdf]

Supplementary Material

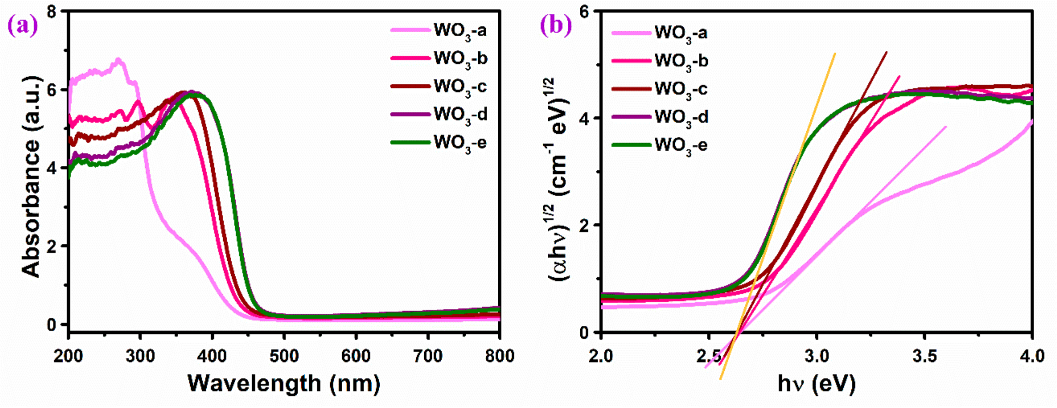

Figure S1 (a) UV-vis absorption spectra and (b) Tauc plots of WO_3 arrays.

The UV-visible light absorption performance of different WO_3 nanoflake arrays is shown in Figure S1. As the coverage of nanoflakes on FTO substrate increases, the light absorption is significantly enhanced. Specially, although the thickness of WO_3-e is obviously higher than that of WO_3-d, the light absorption properties of WO_3-e and WO_3-d are similar, indicating that WO_3-d with the thickness of 3.2 μm can ensure adequate light absorption. According to the corresponding Tauc curve in Figure S1b, the band gap of all the WO_3 arrays is estimated to be the same, about 2.64 eV.

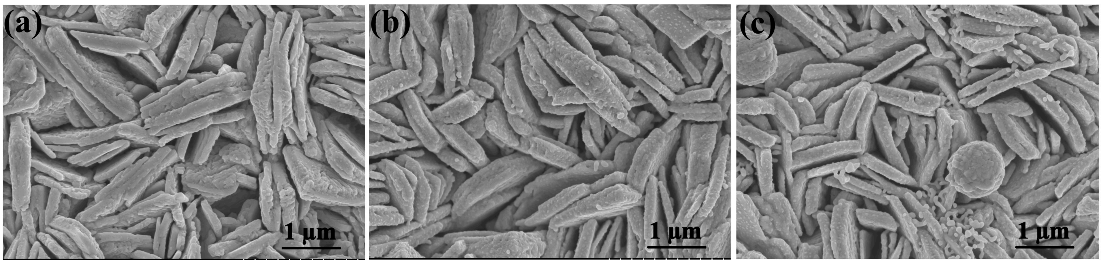

Figure S2 SEM images of $WO_3/BiVO_4$ composite arrays, (a) $WO_3/BiVO_4$-5, (b) $WO_3/BiVO_4$-10, (c) $WO_3/BiVO_4$-20.

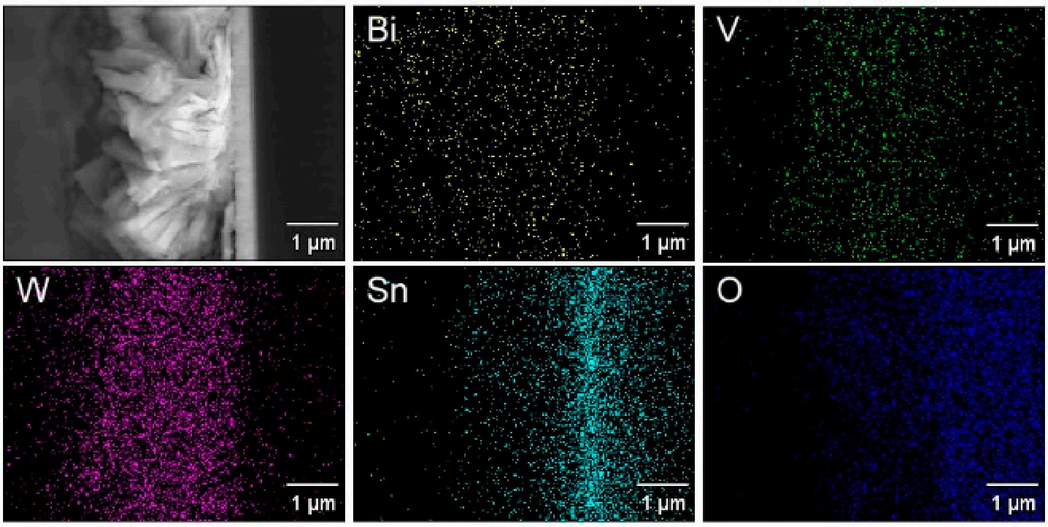

Figure S3 Cross-sectional view SEM image of $WO_3/BiVO_4$-10 and the corresponding elemental mapping.

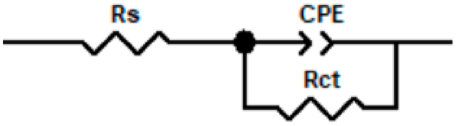

Figure S4 The equivalent circuit of the electrochemical impedance spectra
